# Do extracellular vesicles have specific target cells?; Extracellular vesicle mediated embryo maternal communication

**DOI:** 10.3389/fmolb.2024.1415909

**Published:** 2024-07-16

**Authors:** Keerthie Dissanayake, Kasun Godakumara, Subhashini Muhandiram, Suranga Kodithuwakku, Alireza Fazeli

**Affiliations:** ^1^ Institute of Biomedicine and Translational Medicine, University of Tartu, Tartu, Estonia; ^2^ Institute of Veterinary Medicine and Animal Sciences, Estonian University of Life Sciences, Tartu, Estonia; ^3^ Department of Anatomy, Faculty of Medicine, University of Peradeniya, Peradeniya, Sri Lanka; ^4^ Department of Animal Science, Faculty of Agriculture, University of Peradeniya, Peradeniya, Sri Lanka; ^5^ Division of Clinical Medicine, School of Medicine and Population Health, University of Sheffield, Sheffield, United Kingdom

**Keywords:** extracellular vesicles, embryo implantation, zinc finger protein 81, target specificity, endometrial epithelial cells, trophoblast cells

## Abstract

Extracellular vesicles (EVs) serve as messengers for intercellular communication, yet the precise mechanisms by which recipient cells interpret EV messages remain incompletely understood. In this study, we explored how the origin of EVs, their protein cargo, and the recipient cell type influence the cellular response to EVs within an embryo implantation model. We treated two types of EVs to 6 different recipient cell types and expression of zinc finger protein 81 (ZNF81) gene expression in the recipient cells were quantified using quantitative polymerase chain reaction (qPCR). The proteomic contents of the EV cargos were also analyzed. The results showed that downregulation of the ZNF81 gene was a specific cellular response of receptive endometrial epithelial cells to trophoblast derived EVs. Protein cargo analysis revealed that the proteomic profile of EVs depends on their cell of origin and therefore may affect the recipient cell response to EVs. Furthermore, trophoblastic EVs were found to be specifically enriched with transcription factors such as CTNNB1 (catenin beta-1), HDAC2 (histone deacetylase 2), and NOTCH1 (neurogenic locus notch homolog protein 1), which are known regulators of ZNF81 gene expression. The current study provided compelling evidence supporting the existence of EV specificity, where the characteristics of both the EVs and the recipient cell type collectively contribute to regulating EV target specificity. Additionally, EV protein cargo analysis suggested a potential association between transcription factors and the specific functionality of trophoblastic EVs. This *in vitro* embryo implantation model and ZNF81 read-out provides a unique platform to study EV specific functionality in natural cell-cell communication.

## 1 Background

Extracellular vesicles (EVs) are small, phospholipid-bilayered vesicles released by nearly all cells into their surrounding extracellular environment. Emerging evidence suggests that EVs play a crucial role in mediating communication among cells, organisms and even different species (Ju et al., 2013; Sanwlani et al., 2020; Berumen Sánchez et al., 2021; Lizarraga-Valderrama and Sheridan, 2021). EVs constitute a highly heterogeneous population comprising various subtypes such as exosomes, microvesicles (MVs), and apoptotic bodies (Willms et al., 2016; 2018). The distinctions among these subtypes arise from differences in size, biogenesis pathways, and content (Yáñez-Mó et al., 2015). Nevertheless, the term “extracellular vesicles” serves as an umbrella term that encompasses all vesicle types released by cells. Hence, an individual cell appears to generate billions of EVs throughout its lifetime, with a substantial portion seemingly designed for intercellular communication (Auber and Svenningsen, 2022). EVs, as biological nanoparticles, can target neighbouring and distant recipient cells within the extracellular milieu. Although natural EVs are promising drug delivery carriers (Zheng et al., 2022), their cellular specificity is thought to be limited. Thus, researchers have turned towards EV engineering techniques to create therapeutic vesicles. However, given the low toxicity and biocompatibility of natural EVs, there is the potential to harness the inherent tropism of EVs toward certain cell types for drug delivery purposes. Despite increasing interest in the therapeutic and diagnostic potential of EVs, the exact mechanisms governing EV targeting specificity across diverse cell types remain elusive. Nevertheless, one of the fundamental questions that remains to be addressed first is whether EVs are inherently programmed to convey specific signals to particular cells or cell types, or whether they simply target cells randomly. In this study, we used a human embryo implantation model to investigate the effect of EV origin, cargo content, and recipient cell type on the targeting properties of EVs.

Embryo implantation is a rate-limiting and key step in establishing successful mammalian pregnancy (Dekel et al., 2010). It involves three pivotal steps: apposition, where the blastocyst interacts with the endometrium, followed by attachment of the blastocyst to the endometrium; and invasion of the blastocyst into the endometrium to establish the placenta (Kim and Kim, 2017). Effective communication between a high-quality embryo and a receptive endometrium is paramount for successful implantation (Mishra et al., 2021). Importantly EVs are emerging as a key player of embryo maternal communication before implantation (Es-Haghi et al., 2019; Godakumara et al., 2021). Previously we showed that trophoblast cells from embryo can transfer multiple non coding RNA transcripts, including zing finger protein 81 (ZNF-81) to receptive endometrial epithelial cells (EECs) using EVs (Es-Haghi et al., 2019). This process resulted in the downregulation of same transcripts in the endometrial cells. Interestingly, ZNF81 also known as HFZ20 or MRX45, is a potential transcriptional regulator that belongs to the krueppel C2H2-type zinc finger protein family. Mutations in ZNF81 gene have been previously described to be related to intellectual disability (Kleefstra et al., 2004). However, the precise role of ZNF81 gene in embryo implantation remains unknown. Nevertheless, the transfer of ZNF81 transcripts from the trophoblast cells to the endometrium and the subsequent downregulation of the same gene transcription in the endometrium suggest its potential utility as a model for deciphering trophoblastic EV functions in the endometrium. However, in subsequent studies, we demonstrated that trophoblast EV function extends beyond the modulation of specific gene transcripts, as they also affect the entire transcriptome and secretome of EECs (Godakumara et al., 2021; Muhandiram et al., 2023). Importantly, we showed that the transcriptomic and secretome changes induced in EECs were highly specific to trophoblastic EVs, as non-trophoblastic EVs failed to produce the same effects. Further analysis of the EV RNA cargo of trophoblastic and non-trophoblastic cells revealed that coding (mRNA) and non-coding RNA (miRNA) from these two sources are different. According to miRNA target prediction analysis, approximately 9% of the genes that were downregulated in EECs in response to trophoblastic EVs in transcriptomic study were identified as high-confidence targets of miRNAs that were uniquely present in trophoblastic EVs (Godakumara et al., 2021). This suggests that EV origin and other cargo content may play a role in mediating EV specificity. However, more than 90% of the transcriptomic changes in EECs cannot be explained by the EV RNA content alone. Therefore, it is possible that other EV cargo molecules such as proteins and lipids may have a role in EV mediated functional changes in the endometrium.

In the current study, we first studied the dynamics of receptive endometrial epithelial cell response to trophoblast derived EVs using ZNF-81 downregulation as an indicator of trophoblastic EV activity. Then we investigated how trophoblast EVs function across various recipient cells of both endometrial non endometrial origin *in vitro*. Additionally, we explored the effect of EV protein cargo on their specific functions. This study aims to shed light on the underlying mechanisms that dictate EV targeting specificity, paving the way for enhanced therapeutic and diagnostic applications of EVs.

## 2 Materials and methods

### 2.1 Cell culture

The human Choriocarcinoma JAr cell line was used as an analog of trophoblastic cells to isolate EVs. HEK 293 cell line was used as a source of non-trophoblastic EVs and also as a recipient cell. The HEC-1-A cell line was used as an analog of non-receptive endometrial epithelial cells. The human endometrial adenosquamous carcinoma cell line (RL95–2) and Ishikawa cell line were used as analogs of receptive endometrial epithelial cells. The human fetal colonic epithelial cell (FHC) line and non-small cell lung carcinoma cell line (HCC-44) were used as cell lines of non-endometrial origin. The JAr, HEK 293, HEC-1-A, and FHC cell lines were obtained from the American Type Culture Collection (ATCC). The Ishikawa cell line was obtained from the European Collection of Authenticated Cell Cultures, and HCC-44 was obtained from the Leibniz Institute DSMZ (German Collection of Microorganisms and Cell Cultures GmbH). JAr, HEK 293, RL95-2 cells were grown as described previously (Godakumara et al., 2021).

Briefly, JAr cells (ATCC Cat# HTB-144, RRID:CVCL_0360) were routinely maintained in RPMI 1640 medium (Gibco™, Bleiswijk, Netherlands) supplemented with 10% fetal bovine serum (FBS) (Gibco™ 10500064), 1% L-glutamine (Sigma, 59202C, Saint Louis, United States) and 1% penicillin/streptomycin (P/S) (Gibco™ 15,140,122, Bleiswijk, Netherlands) and incubated at 5% CO_2_ at 37°C. HEK 293 cells (ATCC, CRL-3216) were grown in Dulbecco’s modified Eagles medium (DMEM 12–604F, Lonza, Verviers, Belgium) supplemented with 10% FBS, 1% P/S, and 1% L-glutamine in 5% CO_2_ at 37°C. HEC-1-A cell line was (ATCC, HTB-112) maintained in McCoy’s 5A medium (ATCC, 30–2007) supplemented with 10% FBS and 1% P/S and incubated with 5% CO_2_ at 37°C. Ishikawa cells (RRID:CVCL_2529) were routinely maintained in MEM **(**Gibco™, Bleiswijk, Netherlands) supplemented with 2 mM glutamine, 1% nonessential amino acids (NEAA), 5% FBS and 1% P/S in 5% CO_2_ at 37°C. RL95-2 cells (ATCC CRL-1671) were cultured in DMEM medium supplemented with 1% P/S, 5 μg/mL insulin (human recombinant insulin, Gibco, Invitrogen, Denmark), 1% L-glutamine and 10% FBS at 37°C in a 5% CO_2_ atmosphere. FHC cells (ATCC, CRL-1831) were cultured in DMEM-F12 medium (ATCC, 30–2006) supplemented with extra 10 nM HEPES (H3375, Sigma-Aldrich), 10 mg/mL cholera toxin (C8052, Sigma-Aldrich), 5 μg/mL insulin, 5 μg/mL transferrin (T8158, Sigma-Aldrich), 100 ng/mL hydrocortisone (H0888-Sigma-Aldrich), 20 ng/mL human recombinant EGF (PHG0311, Thermo Fisher), 10% FBS and 1% P/S at 37°C in a 5% CO_2_ atmosphere. HCC-44 cells (ACC 534) were cultured in RPMI-1640 medium (Gibco, Scotland) supplemented with 10% FBS and 1% P/S at 37°C in 5% CO_2_. Media renewal for all cell lines was carried out once every 2 days.

### 2.2 EV isolation and characterization

EV isolation and characterization from JAr and HEK 293 cells were carried out as described in our previous publications (Midekessa et al., 2020; Godakumara et al., 2021; Muhandiram et al., 2023). Briefly, JAr and HEK cells were grown until it reached 80% confluency and the cell culture-conditioned media were replaced with EV-depleted FBS containing media. After 24 h of media change, the cell culture supernatants were collected (100 mL each) and sequential centrifugation was performed at 400 × g for 10 min to remove contaminating cells followed centrifugation at 4,000 *g* for 10 min and 10,000 × g for 10 min to remove remaining cellular debris. Then the collected media were concentrated to a final volume of 500 µL using Amicon^®^ Ultra-15 centrifugal filters with a 10 kDa cut-off. Finally, EVs were purified using size-exclusion chromatography (SEC), fractions 7–10 were collected (each fraction was 500 µL in volume) and concentrated again to a total volume of 500 µL using an Amicon^®^ Ultra centrifugal filter device with a 10 kDa cut-off (Reshi et al., 2021). Both the size and concentration of nanoparticles in the EV fractions were measured using Nano particle tracking analyser (Particle Metrix GmbH, Inning am Ammersee, Germany) (Dissanayake et al., 2021a). Further physical characterization was carried out using transmission electron microscopy and western blotting or label free proteomic analysis to detect enrichment of EV protein markers. EVs derived from JAr cells and HEK 293 cells are referred to as JAr EVs and HEK EVs, respectively.

### 2.3 RNA extraction

Following incubation of different recipient cells with EVs for the defined duration and with defined concentration of EVs, the cell culture supernatants were discarded. Cells were washed with Dulbecco’s phosphate-buffered saline without Ca2+ and Mg2+ (DPBS, Verviers, Belgium) and total RNA was extracted using phenol-chloroform based RNA extraction as described previously (Godakumara et al., 2023). TRIzol reagent (TRIzol^®^ reagent; Invitrogen, Inchinnan, UK) was added directly to the cell layer and incubated with cells for 10 min to completely dissociate the nucleoprotein complexes. Then, 300 µL of chloroform for 1 mL of TRIzol was added to the samples, vigorously shaken for 15 s and centrifuged at 12,000 × g for 15 min at 4°C. When the phases were separated, the aqueous phase containing the RNA was removed, and the RNA was precipitated using 500 µL of isopropanol at room temperature for 20 min. To increase the efficiency of RNA extraction, 10 µg of glycogen (UltraPure™ Glycogen, 10814–010, Thermo Fisher Scientific, Bleiswijk, Netherlands) was added to the mixture. Once the precipitation was complete, the samples were centrifuged at 18,000 × g for 30 min at room temperature to pellet the RNA. The RNA pellets were washed three times in 70% ethanol and eluted in 20 µL of nuclease-free water. Quantity and the quality of RNA were measured using NanoDrop™ 2000 spectrophotometer (Thermo Fisher Scientific, United States).

### 2.4 cDNA synthesis and quantitative real-time PCR (RT-qPCR) validation

RNA reverse transcription was performed using FIREScript RT cDNA Synthesis mix™ and using a mixture of oligo and random primers (Solis BioDyne, Estonia). For ZNF81 copy number quantification, cDNA product was amplified by RT-PCR using HOT FIREPol^®^ EvaGreen^®^ qPCR Supermix (Solis BioDyne, Estonia) and QuantStudio 12K Flex™ real-time PCR system (ThermoFisher Scientific). The PCR instrument settings: 95°C for 15 min followed by 40 cycles of 95°C for 20 s, 60°C for 20 s, 72°C for 20 s. The copy number of ZNF81 gene transcript was calculated by absolute quantification method and as described earlier (Es-Haghi et al., 2019).

### 2.5 Immunofluorescence

The subcellular localization of ZNF81 was analyzed by immunostaining JAr and RL95-2 cells. First, 10^4^ JAr and RL95-2 cells were seeded in 8 well chamber slides and allowed to grow overnight. Cells were washed with DPBS and fixed using 4% paraformaldehyde (PFA) in PBS for 20 min. PFA was removed from cells by washing three times in PBS and cells were permeabilized with 0.1% Triton X-100 for 15 min at room temperature. Samples were washed 3 times with PBS/0.1% bovine serum albumin (BSA) and blocked for 30 min at room temperature using 4% goat serum (Jackson ImmunoResearch Laboratories, United Kingdom) in PBS. The samples were then incubated with ZNF81 antibody (Thermo Fisher Scientific Cat# PA5-51625, RRID:AB_2650354) diluted in PBS with 4% goat serum overnight at 4°C (1:500). Aleza flour™ 594 goat anti-rabbit secondary antibody (1:1000, Thermo Fisher Scientific Cat# A-11012, RRID:AB_2534079) was used to bind the primary antibody. Nuclei were counterstained with DAPI and imaged using confocal laser scanning microscope (FV1000 system OVF 10278, Olympus). A well containing secondary antibody alone was used as a negative control to detect the background fluorescence and to fix the exposure time. The target specificity of ZNF 81 antibody was validated with the ZNF81 synthetic peptide (Sigma- Aldrich, APREST74142) and immunofluorescent staining.

### 2.6 Western blotting

Total protein concentrations in cell lysates were determined by the Bradford assay (Pierce 23246) to normalize the total protein loading of all samples. The samples were then separated using 12% SDS-PAGE, and transferred to a polyvinylidene difluoride (PVDF) membrane. The membranes were blocked in 5% milk in phosphate-buffered saline with 0.1% Tween 20 (PBST) and incubated overnight with primary antibodies against, β-actin (1:5000, Proteintech Cat# 20536-1-AP, RRID:AB_10700003) and ZNF81 (1:1000, Santa Cruz Biotechnology Cat# sc-81429, RRID:AB_2220247) in 5% milk-PBST solution. Following this, the membranes were exposed to horseradish peroxidase-conjugated goat anti-mouse (1:10000, Santa Cruz Biotechnology Cat# sc-516102, RRID:AB_2687626) or goat anti-rabbit secondary antibody (1: 10000, G Thermo Fisher Scientific Cat# G-21234, RRID:AB_2536530) for 2 h at room temperature. Between each incubation step, the membranes were washed thrice for 5 min each in PBST. Protein bands in PVDF membranes were visualized using ECL Select™ Western Blotting Detection Reagent (GE Healthcare, Buckinghamshire, United Kingdom) and ImageQuant™ RT ECL Imager (GE Healthcare, Buckinghamshire, United Kingdom).

### 2.7 Liquid chromatography tandem mass spectrometry (LC/MS/MS)

Proteins in JAr and HEK EV **s**amples were precipitated with trichloroacetic acid deoxycholate (TCA-DOC) precipitation overnight and as described earlier with few modifications (Muhandiram et al., 2023). The cell pellets were solubilized in 7 M urea, 2 M thiourea, 100 mM ammonium bicarbonate (ABC), 20 mM methylamine buffer. Protein reduction was performed with 5 mM dithiothreitol (DTT) by incubating 1 h at room temperature. Protein alkylation was performed with 10 mM chloroacetamide by incubating 1 h at room temperature in the dark. Next, protease LysC (Wako) was added to an enzyme:substrate ratio (E:S) of 1:50 and the samples were incubated for 1 h at room temperature. Samples were then diluted five times with 100 mM ABC, trypsin (Sigma Aldrich) was added to 1:50 E:S ratio and incubated overnight at room temperature. After digestion, samples were acidified with trifluoroacetic acid (TFA) to a concentration of 1%, and samples were desalted on in-house made C18 StageTips. Samples were reconstituted in 0.5% TFA and peptide concentrations were determined with a Pierce colorimetric peptide assay (Thermo Fisher Scientific). 1 ug of peptides were then subjected to LC/MS/MS analysis.

Five hundred nanograms of protein were injected to an Easy-nLC 1000 system (Thermo Scientific) and MS analysis was performed as described previously with few modifications (Reshi et al., 2023). The sample was eluted at 250 nL/min from the trap to a 75 µm ID ×50 cm emitter-column (New Objective) packed with C18 material (3 μm, 300 Å particles, Dr Maisch). The separating gradient was 2%–35% B 60 min and 40%–100% B 5 min (A: 0.1% formic acid (FA), B: 80% ACN + 0.1% FA). Eluted peptides were sprayed to a Q Exactive Plus (Thermo Fisher Scientific) quadrupole-orbitrap mass spectrometer (MS) using nano-electrospray ionization at 2.4 kV (applied through liquid-junction). The MS was operated with a top-10 data-dependent acquisition strategy. Briefly, one 350–1400 m/z MS scan at a resolution setting of R = 70,000 at 200 m/z was followed by ten higher-energy collisional dissociation fragmentation (normalized collision energy of 26) of 10 most intense ions (z: + 2 to + 6) at R = 17,500. MS and MS/MS ion target values were 3e6 and 5e4 with 50 ms injection time. Dynamic exclusion was limited to 30 s.

### 2.8 Differential Protein expression and Bioinformatics analysis

MS raw files were processed with the MaxQuant software package (version 1.6.15.0) as described previously with few modifications (Piibor et al., 2023). Methionine oxidation, and protein N-terminal acetylation were set as variable modifications, while cysteine carbamidomethylation was defined as a fixed modification. Search was performed against UniProt (www.uniprot.org) *Homo sapiens* and *Bos taurus* proteome database using the tryptic digestion rule (allowing cleavages after lysine and arginine without proline restriction). Only identifications with at least one peptide of 7 or more amino acids in length, with up to 2 missed cleavages, were considered acceptable. Transfer of identifications between runs based on accurate mass and retention time was enabled. Label-free normalization was performed using the MaxQuant LFQ algorithm. The LFQ ratio count, which represents the number of quantified peptides necessary for reporting a protein intensity, was set to 2. Peptide-spectrum match and protein false discovery rates (FDR) were maintained below 1% using a target-decoy approach. All other parameters were set to their default values.

The raw data files were analysed using MaxQuant software to obtain protein identifications and their respective label-free quantification values using in-house standard parameters. The proteomic data analysis was performed using LFQ-analyst (Shah et al., 2020) platform. In summary, data was normalized based on the assumption that majority of proteins do not change between two EV types. Initially, three classes of proteins: contaminant proteins, reverse sequences and proteins identified “only by site” were filtered out. Proteins that have been only identified by a single peptide and proteins not identified/quantified consistently in same condition have been removed as well. The LFQ protein intensity values were log2 transformed. Imputation of the missing values were performed by “Missing not At Random” (MNAR) method where random draws from a left-shifted Gaussian distribution were used. Protein-wise linear models combined with empirical Bayes statistics were used for the differential expression analyses. A cutoff of the adjusted *p*-value of 0.05 (Benjamini-Hochberg method) along with a log2 fold change of 1 has been applied to determine differentially enriched protein in pairwise comparison. The mass spectrometry proteomics data have been deposited to the ProteomeXchange Consortium via the PRIDE partner repository with the dataset identifier PXD050024.

Functional annotation and pathway over representation analysis were performed using the Database for Annotation, Visualization and Integrated Discovery (DAVID Bioinformatics platform) (Huang et al., 2009; Sherman et al., 2022). All relevant gene names were submitted to the DAVID Bioinformatics platform for Kyoto Encyclopedia of Genes and Genomes **(**KEGG**)** pathway enrichment and gene ontology (GO) enrichment analysis. Adjusted *p*-value < 0.05 considered as statistically significant.

### 2.9 *In silico* transcription factors (TF) analysis

A curated list of human TFs and their targets were collected from the comprehensive TF/Target database, hTFtarget (PMID: 32858223) (Zhang et al., 2020). TFs with the potential to target ZNF81 were filtered out and the presence and expression of these TFs in the EVs were collected from the relative enrichment datasets. TFs with significant (adjusted *p*-value < 0.05) enrichment or depletion were considered for further considerations.

### 2.10 Experimental design

#### 2.10.1 Dynamics of ZNF81 gene expression in RL95-2 cells in response to JAr EVs

The dynamics of ZNF81 gene expression in receptive endoemtrial epitheilal cells in response to trophoblast derived EVs were determiend as a function of time and dose. First, RL95-2 cells were co-incubated with 10^8^ particles/mL of JAr cell derived EVs (200:1 EV:cell ratio) in a time gradient consisting of 30 min, 2 h, 4 h, 8 h, and 24 h time points. RL95-2 cells were also co-incubated with JAr EVs in a concentration gradient (1 × 10^1^ to 1 × 10^10^ EVs to 5 × 10^5^ cells) for 24 h to investigate the effect of JAr EVs on the expression of ZNF81 in RL95-2 cells.

#### 2.10.2 Determining the ZNF81 subcellular localization

Subcellular localization of the ZNF81 protein in JAr and RL95-2 cells was determined using immunofluorescence to better understand the potential functions of the ZNF81 protein.

#### 2.10.3 Determining how estrogen and progesterone hormones affect ZNF81 protein expression in RL95-2 cells

Endometrial RL95-2 cells were treated with estrogen and progesterone hormone combinations that mimics follicular and luteal phases of the menstrual cycle. RL95-2 cells were grown in 6 well plates until 70% confluency and existing medium were replaced with phenol red free DMEM F12 medium supplemented with 5% P/S, 5 μg/mL insulin (serum free) and incubated for 24 h in 5% CO_2_ at 37°C. After 24 h, RL95-2 cells were supplemented with estrogen and progesterone hormone combinations that mimics the luteal phase (10 nM estrogen 1 µM progesterone) and proliferative phase (1 µM estrogen 100 nM progesterone) of the menstrual cycle (Sarhan et al., 2013; Gurung et al., 2020; Poh et al., 2021) in phenol red free DMEM F12 medium supplemented with 5% EV depleted charcoal stripped FBS, 1% P/S and 5 μg/mL insulin for 24 h time. Next, cells were washed with ice cold PBS once and total protein were isolated by lysing the cells in 250 μL of radio immunoprecipitation buffer (RIPA, Pierce, Rockford, IL) with protease inhibitors. Cell lysate was centrifuged at 15,000 *g* for 5 min at 4°C, supernatants were collected and stored at −80°C.

#### 2.10.4 Specificity of ZNF81 gene expression in receptive endometrial epithelial cells in response to trophoblast derived EVs

Six cell lines, two representing human receptive endometrial epithelial cells (RL95-2 and Ishikawa cells), HEC-1A representing human non receptive endometrial epithelial cells and 3 representing human non-endometrial cells (FHC, HCC-44 and HEK 293) were chosen as recipient cells and cultured for the EV supplementation experiments. When cells reached 80% confluency, they were washed with Dulbecco’s phosphate-buffered saline without Ca^2+^ and Mg^2+^ (DPBS, Verviers, Belgium). The cells were then supplemented with EVs derived from trophoblast analog JAr cells at a concentration of 1 × 10^8^ particles/mL in EV-depleted FBS containing medium for 4 h and 24 h. HEK EVs were used as a control EV source and recipient cells were treated in similar concentrations for 4 h and 24 h. PBS treated controls were also used as untreated controls. After incubation, cells were washed and cellular RNA was isolated from each recipient cells for ZNF81 gene expression analysis.

#### 2.10.5 Determining the proteomic profile of JAr and HEK EV protein cargo and transcriptomic factor analysis

JAr and HEK EV proteomic analysis was performed using LC-MS/MS to better understand the EV protein signals that might contribute to specific transcriptomic and proteomic changes of the endometrial epithelial cells. Potential transcriptomic factors in JAr EVs that are responsible for ZNF81 downregulation in the endometrial cells were further analyzed *in silico*.

#### 2.10.6 Statistical data analysis

Data were analysed using GraphPad Prism v8.4.3. The normality and the homogeneity of variance of the data were tested with Shapiro-Wilk test and Levene’s test respectively. Unpaired *t*-test was performed for comparison between two groups. For multiple group comparisons, one-way ANOVA or Tukey’s Honestly Significant Difference (HSD) test was used. Experiments were conducted in triplicates and data were presented as mean plus/minus standard deviation (mean ± SD). In all analyses, *p* < 0.05 is considered statistically significant.

## 3 Results

### 3.1 Dynamics of cellular response of receptive endometrial epithelial cells to trophoblastic EVs

EVs isolated from JAr and HEK293 cells were characterized using nanoparticle tracking analysis, western blotting or LC-MS/MS methods. Previously, we reported the results of EVs characterization (Es-Haghi et al., 2019; Midekessa et al., 2020).

**FIGURE 1:**
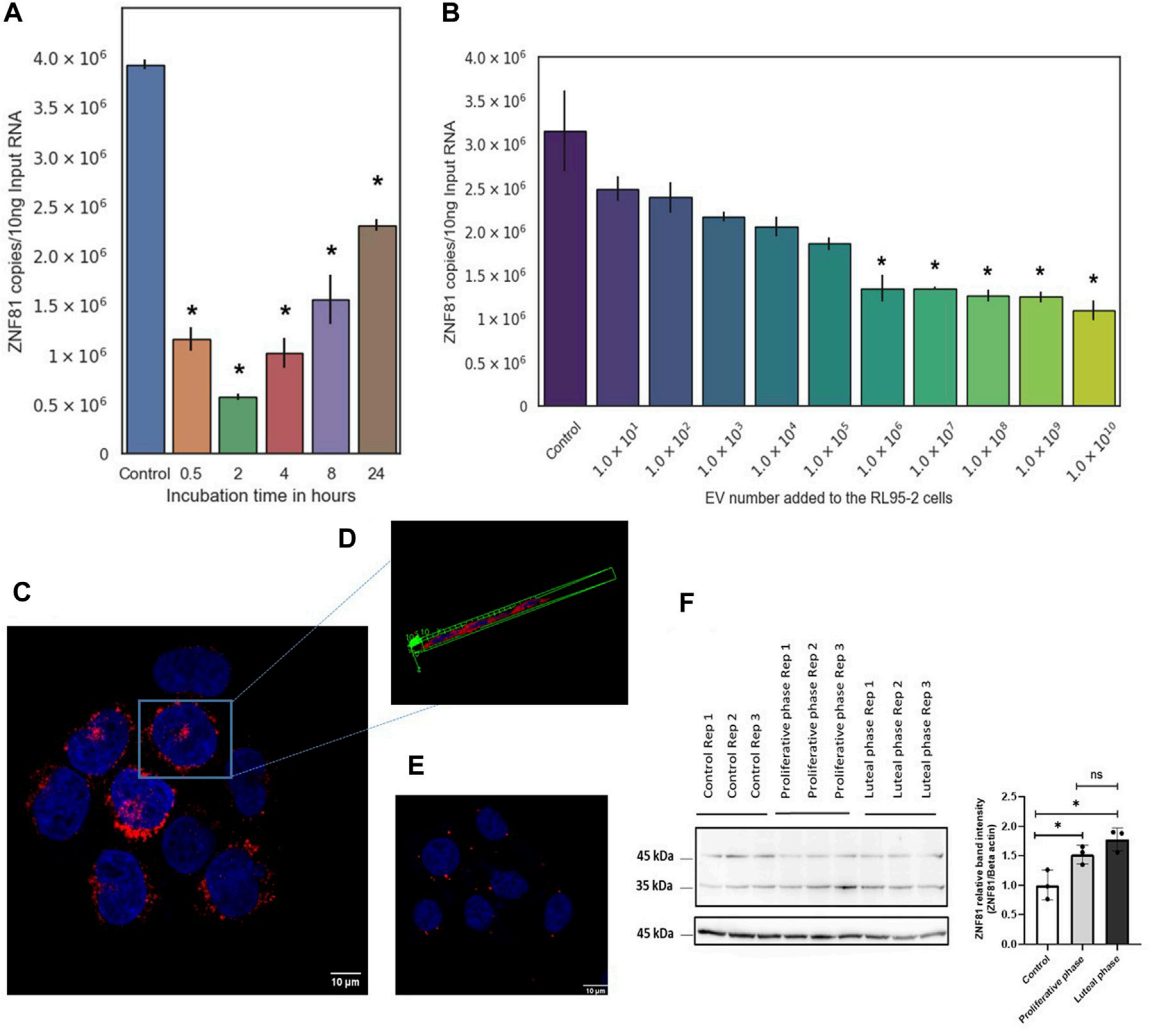
ZNF81 gene expression in receptive endoemtrial epithelial cells was primed by trophoblastic EVs and major steroid hormones of the menstrual cycle. RL95-2 cells were co-incubated with JAr EVs in a **(A)** time gradient (30 min, 2 h, 4 h, 8 h and 24 h with 200:1 EV:cell ratio) and in a **(B)** concentration gradient (1 × 10^1^ to 1 × 10^10^ EVs to 5 × 10^5^ RL 95–2 cells) to investigate the effect of JAr EVs on the expression of ZNF81 in RL95-2 cells. ZNF81 subcellular localization was studied using immunoflourascence staining. **(C)** JAr cells showing ZNF 81 protein expression outside nucleus and in vesicles. **(D)** Three dimensional confocal scanning of JAr cells showing ZNF 81 perinuclear subcellular localization **(E)** RL95-2 cells showing ZNF 81 subcellular localization outside nucleus and in vesicles **(F)** ZNF81 protein expression in hormone treated RL95-2 cells. ZNF 81 protein was significantly higher in hormone treated groups compared to the untreated controls (*p* < 0.05). No significant differences of ZNF81 protein expression was detected between luteal and proliferative phase mimics (*p* > 0.05). * Asterisk indicates *p* < 0.05. All the experiments were conducted in three biological replicates.

Then, RL95-2 cells were co-incubated with JAr EVs in a time gradient (30 min, 2 h, 4 h, 8 h, and 24 h with 200:1 EV:cell ratio) and a concentration gradient (1 × 10^1^ to 1 × 10^10^ EVs to 5 × 10^5^ cells) to investigate the effect of JAr EVs on the expression of ZNF81 in RL95-2 cells. Significant downregulation of ZNF81 expression (*p* < 0.05) was observed in all the observed time points in RL95-2 cells treated with 1 × 10^6^ to 1 × 10^10^ EVs (1: 2 to 1: 2 × 10^4^ EV:Cell ratio). This suggested that ZNF81 expression in RL95-2 changes even when incubated with very low concentration of JAr EVs for a very short time period (Figures 1A,B).

However, the role of ZNF81 in the human endometirum has not yet been described. Therefore, we studied its subcellular localization and responsiveness to estrogen and progesterone (main steroid hormones that regulate menstrual cycle changes of the endometrium) to better understand its functional role in the endometrium.

### 3.2 The protein encoded by ZNF81 gene has a perinuclear localization and was responsive to estrogen and progesterone hormone combinations

The results of the confocal microscopy analysis showed that ZNF 81 protein is primarily found in perinuclear cytoplasmic areas, with a significant presence in perinuclear intracellular vesicles and a weaker presence in the cytoplasm in both JAr and RL95-2 cells (Figures 1C–E). ZNF81 protein expression was significantly higher in luteal and proliferative phase mimics compared to untreated controls (*p* < 0.05). However, no significant differences in ZNF81 protein expression were detected between luteal and proliferative phase mimics (*p* > 0.05). Therefore, ZNF81 protein seems to be affected by presence or absence of estrogen and progesterone hormones and not by their alterations as it may happen during menstrual/reproductive cycle in the endometrium (Figure 1F).

### 3.3 Trophoblastic EV function was specific towards receptive endometrial epithelial cells

**FIGURE 2:**
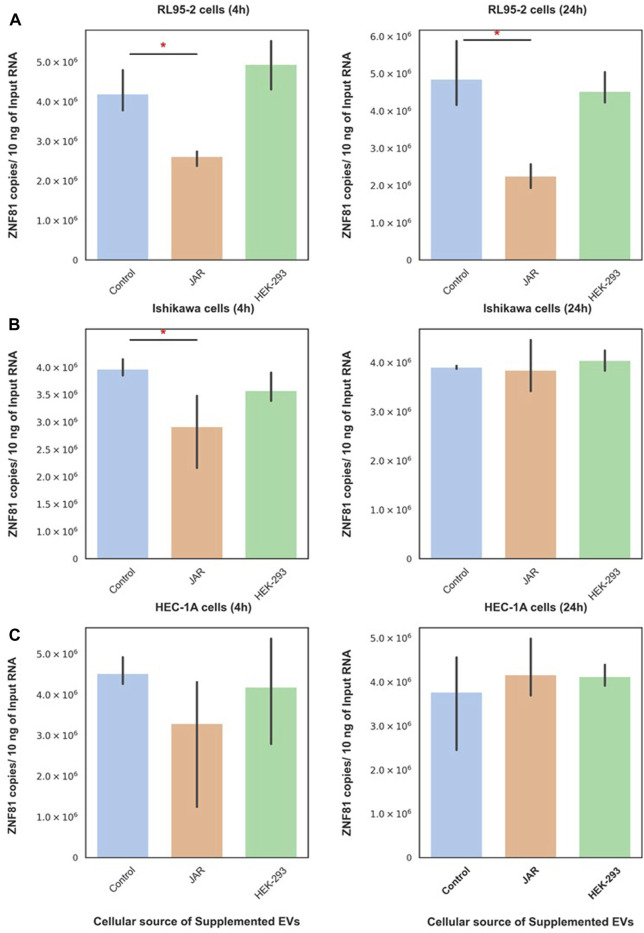
ZNF81 downregulation was a specific response of receptive endometrial cells to trophoblastic EVs. ZNF81 gene expression in 3 different endometrial epithelial cell lines (RL95-2, Ishikawa and HEC-1A) in response to JAr EVs and HEK EVs at 4 h and 24 h **(A)** RL-95 cells (4 h) and RL-95 cells (24 h), **(B)** Ishikawa (4 h) and Ishikawa (24 h), **(C)** HEC-1A cells (4 h) and HEC-1A (24 h). * Asterix indicates *p* < 0.05. All the experiments were conducted in three biological replicates.

JAr EVs downregulated ZNF-81 gene expression in RL95-2 cells at both 4 h and 24 h (Figure 2A). Moreover, the supplementation of Ishikawa cells with JAr EVs downregulated the ZNF81 gene expression at 4 h, but not at 24 h (Figure 2B). Interestingly, this downregulation of ZNF-81 gene expression was not observed in HEC-1A (Figure 2C). Furthermore, supplementation with HEK EVs did not alter the ZNF-81 expression in any of the reproductive cell lines.

Subsequently, JAr and HEK EVs were supplemented to non-endometrial/non-reproductive cells (FHC, HEK-293, and HCC-44) to investigate the specificity of endometrial cells in response to trophoblast-derived EVs. Interestingly, neither trophoblastic EVs (JAr EVs) nor non-trophoblastic EVs (HEK EVs) altered ZNF81 gene expression in FHC, HEK-293 or HCC-44 cells at 4 h or 24 h. The results of this experiments indicated that the trophoblast EV function is recipient cell dependent and trophoblast EVs can potentially mediate communication only between embryo and receptive endometrial cells.

### 3.4 Trophoblastic cell derived EVs had a unique protein cargo

**FIGURE 3:**
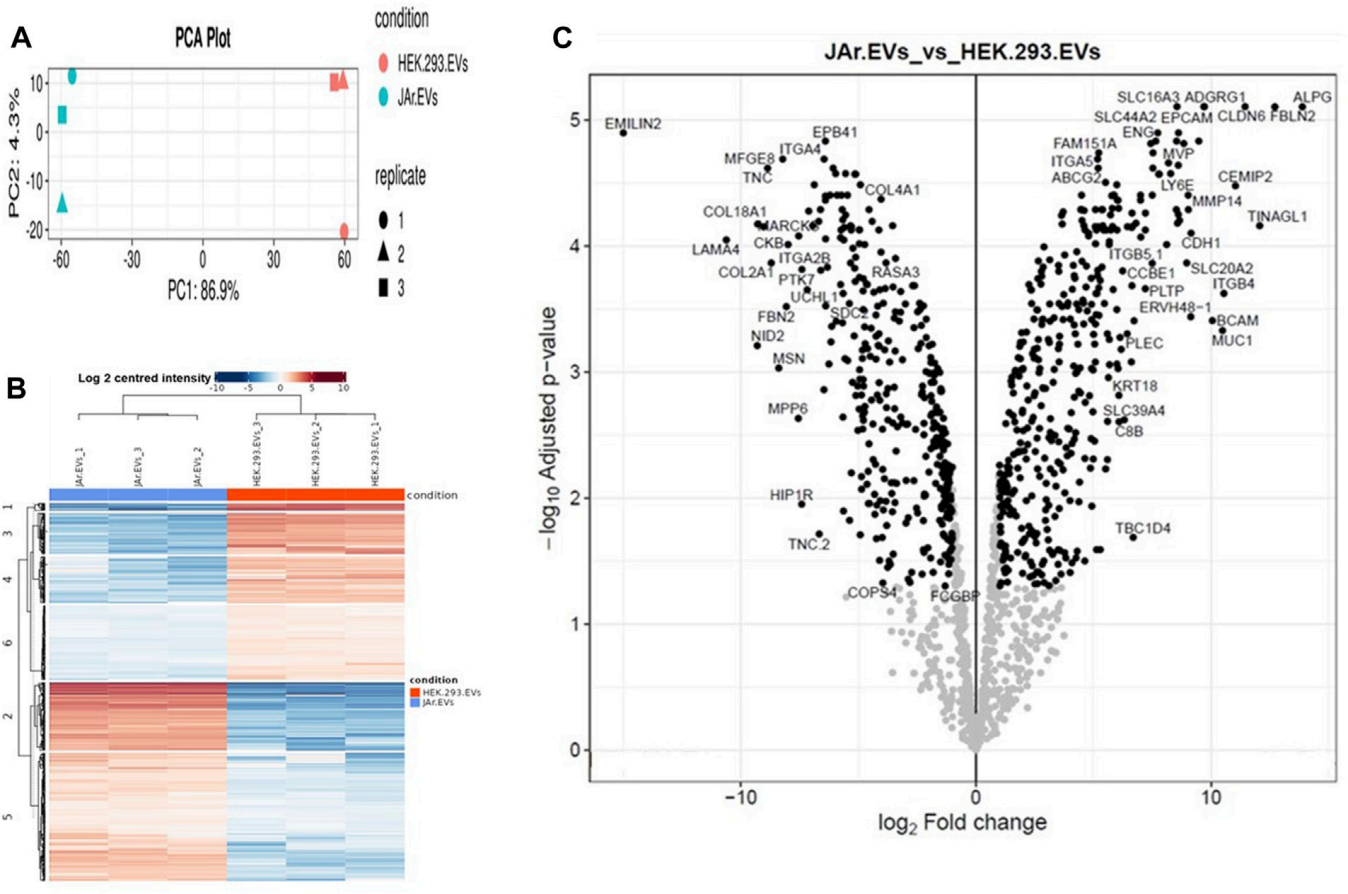
The JAr EVs proteomic profile compared to HEK EVs. **(A)** The principal component analysis (PCA) indicates the separation of the JAr and HEK EV proteomic cargo profile along the principle component 1 and 2. **(B)** The heatmap indicates hierachichal clustering of all differentially expressed proteins (rows) across all 6 samples (columns) where the difference between the JAr EV and HEK EV groups is apparent. All the differentially enriched proteins in JAr EVs were categorized into 6 clusters. **(C)** Volcano plot illustrating the up and downregulated proteins in JAr EVs compared to HEK EVs. All the experiments were conducted in three biological replicates.

To gain insight into the effect of EV protein cargo on its specific functionality in the endometrium, we analyzed JAr and HEK EV protein cargo using LC-MS/MS (Supplementary Table S1). In total, 1448 proteins were reproducibly quantified in both EV types. Interestingly, the PCA plot and heatmap showed a clear segregation of the JAr EV samples compared to the HEK EV samples (Figures 3A,B). There were 740 proteins differentially expressed between the two groups (log2 fold change ≥1 and adjusted *p*-value < 0.05) (Figure 3C). Out of them, 397 proteins were enriched and 343 proteins were depleted and in JAr EVs compared to HEK EVs (Supplementary Table S2).

Proteins identified as specially enriched in JAr EVs compared to HEK EVs were subjected to functional annotation, KEGG and GO pathway enrichment analysis.

**FIGURE 4:**
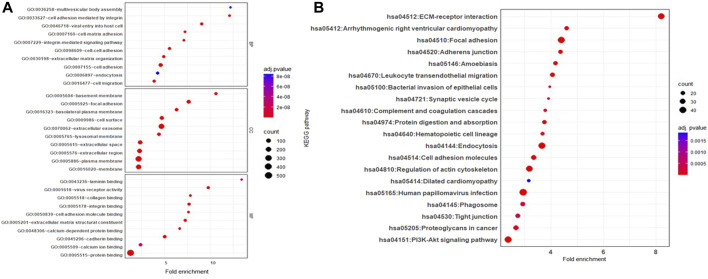
GO and KEGG pathway analysis of proteins specifically enriched in JAr EVs compared to HEK EV **(A)** GO pathway analysis of ten remarkably enriched items in the biological processes (BP), cellular components (CC), and molecular functions (MF) respectively. **(B)** KEGG analysis of differentially expressed proteins between JAr and HEK EVs. Top 20 enriched pathways/conditions were shown.

Proteins unique to JAr EVs demonstrated enrichment of GO biological processes, such as “extracellular matrix organization,” “integrin mediated cell signaling,” and “cell-cell adhesion.” Moreover, GO molecular functions such as “laminin binding,” “virus receptor activity,” “collagen binding” and “cell adhesion molecule binding” were highly enriched in JAr EVs compared to HEK EVs (Figure 4A). According to KEGG pathway analysis, pathways such as “ECM receptor-interaction,” “focal adhesion,” and “adherens junctions, etc. were notably highlighted in JAr EVs compared to HEK EVs (Figure 4B). All GO and KEGG pathways enriched in proteins unique to JAr EVs are shown in Supplementary Tables S3–6)

Proteins highly enriched in JAr EVs compared to HEK EVs were implicated in cell-cell adhesion (e.g., FBLN2, ITGB4, BCAM, CDH1, ITGB5) (Chang et al., 2017; Meng et al., 2020; Ma et al., 2021; Bhattacharyya et al., 2022), early embryo development/differentiation (e.g., FOLR1, ERVW-1, CLDN6, EPCAM) (Moriwaki et al., 2007; Nagao et al., 2009; Strandgaard et al., 2017; C.West et al., 2022), and endometrial receptivity (e.g., ENG, MMP2, MMP14) (Arthur et al., 2000; Cabral-Pacheco et al., 2020) suggesting a potential role of trophoblastic EV protein cargo in the embryo implantation process.

### 3.5 Trophoblastic cell derived EVs were enriched with TFs known to regulate the expression of ZNF81

**FIGURE 5:**
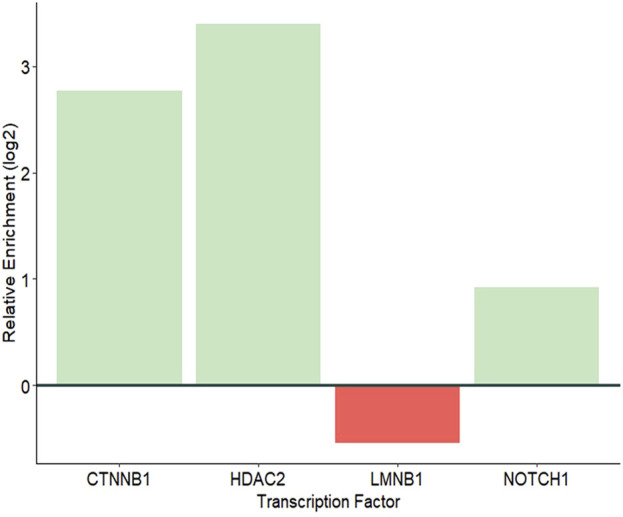
Relative enrichment of TFs that may regulate the expression of ZNF81.

Eight known TFs were observed in the proteomes of JAr EVs. Of these, 4 TFs, CTNNB1, HDAC2, LMNB1 and NOTCH1 are capable of regulating ZNF81 gene expression. CTNNB1, HDAC2 and NOTCH1 were significantly enriched in JAr EVs compared with HEK EVs (Figure 5). The results showed a potential role of trophoblast EV protein cargo molecules in ZNF81 gene expression in the endometrium.

## 4 Discussion

An essential yet inadequately explored aspect of EV biology is whether EVs produced by individual or similar groups of cells are engineered to influence specific cell types, tissues, or organs. In other terms, there is a lack of understanding regarding whether natural EVs exhibit preference for specific cells in multicellular environments. If EVs are cell-specific, this implies that they are secreted with a specific address on them. However, the current body of research lacks comprehensive investigations framed around this specific question. The specificity of EVs also lacks a clear definition in literature. Recent studies have proposed that some sort of EV tropism may exist in terms of their uptake (Toda et al., 2015; Jurgielewicz et al., 2020) or the changes they induce in selected recipient cells (Hoshino et al., 2015; Godakumara et al., 2021; Hasan et al., 2021). If EV specificity exists, it may be linked to various factors, including, but not limited to EV origin (Dissanayake et al., 2021b; Hagey et al., 2023), subpopulation (Bruno et al., 2017; Wahlund et al., 2017), surface charge (Kaddour et al., 2020), cargo content (Hoshino et al., 2015; Godakumara et al., 2021), and recipient cell type (Czernek et al., 2015). In this study, we aimed to determine whether EVs exhibit specificity in targeting particular cells and to explore the potential factors governing this specificity. Addressing this knowledge gap will help in the effective use of EVs as both therapeutic and diagnostic tools.

In our previous study, we demonstrated that trophoblast-derived EVs could downregulate ZNF81 expression in receptive endometrial epithelial cells (Es-Haghi et al., 2019). In this study, we examined the dynamics of embryo-endometrium communication as a function of dose and time using ZNF81 downregulation as a trophoblastic EV activity. Our findings indicate that trophoblast EVs induce changes in ZNF81 expression in receptive EECs as early as 30 min post-EV exposure, with an effective dose as low as approximately 50 EVs per cell. This rapid transcriptional response aligns with our previous reports. Despite the seemingly low EV-to-cell ratio, our prior work underscores that such a minimal number of EVs per cell is sufficient for their functional impact (Godakumara et al., 2023). Previous studies also suggest that cellular responses to EVs can be dose and time dependent (Franzen et al., 2014; Hagey et al., 2023); however, there are discrepancies in the minimum time and dose of EVs required for their uptake or functionality within recipient cells (Emam et al., 2019; Jurgielewicz et al., 2020; O’Brien et al., 2022). Compared with other studies, trophoblast EVs require a short time and low dose to induce cellular responses indicating that some form of tropism may exist between trophoblast EVs and receptive endometrial epithelial cells (Tabak et al., 2018; Hagey et al., 2023).

ZNF81 gene or protein is not previously reported as playing any role in embryo implantation. The protein encoded by ZNF81 is known as a potential DNA binding transcription factor (Kleefstra et al., 2004). Zinc finger family proteins are among the most abundant groups of proteins expressed during early embryogenesis in mammals (Su et al., 2021). However, the exact subcellular localization of ZNF81 and its functional role in the human endometrium have not yet been reported. Here, we demonstrated that the ZNF81 protein shows perinuclear cytoplasmic localization in both trophoblasts and receptive endometrial epithelial cells. Furthermore, ZNF81 protein expression was sensitive to estrogen and progesterone hormone treatments. However, we could not see significant changes of ZNF81 protein expression between luteal and proliferative mimics of RL95-2 cells. This may be due to the fact that RL95-2 cells are already receptive in nature. The perinuclear localization of ZNF81 suggests its possible role as a transcription factor while the hormone responsiveness of ZNF 81 protein suggested that it could be differentially regulated *in vivo* in different phases of the menstrual cycle. Thus, understanding the binding partners and studying the changes of this protein further in the human endometrium during the menstrual cycle will be of interest to decipher the potential functions of the ZNF81 protein in the context of embryo implantation regulation.

As ZNF81 protein showed some relevance to endometrial function in the hormone study, we further investigated whether ZNF81 downregulation is specific to the endometrium. For that, trophoblastic EVs were incubated with multiple recipient cells, including those of both endometrial and non-endometrial origin. We stimulated different recipient cells with a single dose of trophoblastic EVs and detected ZNF81 downregulation at two time points: 4 h and 24 h. The ZNF81 downregulation occurs exclusively in receptive endometrial epithelial cells, highlighting the specificity of trophoblastic EV functionality. However, ZNF81 down regulations in Ishikawa cells were shown only in 4 h time point in contrast to RL95-2 cells that shows the response persisting up to 24 h. In a recent study, we examined the dynamics of transcriptomic changes induced by trophoblast EVs in RL95-2 cells and observed transcriptomic changes at 30 min, 4 h, and 48 h. The highest activity was noted at the 4-h time point, implying a greater extent of specific transcriptomic alterations. By the 48-h time point, the activities showed a return to baseline levels, indicating that the effects of EVs wane over time (Godakumara et al., 2023). Therefore, a single dose of EVs may not induce a permanent gene transcription response. We can infer that in Ishikawa cells, EVs also showed the highest transcription activity at 4 h, which then diminished by 24 h. The differences between RL95-2 cells and Ishikawa cells may be attributed to the presence of different receptors on the cell surface for EVs or the use of different EV internalization routes, which can affect EV activity (Feng et al., 2010; Parada et al., 2021). Interestingly, trophoblastic EVs failed to induce changes in ZNF81 gene expression in non-receptive endometrial epithelial cells and non-endometrial cells. On the other hand, non trophoblastic EVs (HEK EVs) did not induce changes in ZNF81 gene expression in any of the cells. These observations imply the existence of specific mechanisms governing the interaction between trophoblastic EVs and receptive endometrial cells. Previous literature provides evidence of EV tropism toward certain cell types, although the precise mechanisms remain unknown. Examples include the preferential trafficking of dendritic cell-derived EVs to immunological sites (Wiklander et al., 2015) and tumor-derived EVs exhibiting generalized tropism towards neoplastic tissues (Garofalo et al., 2019). Hence, EVs have shown tropism especially towards the same cell of origin (Toda et al., 2015; Jurgielewicz et al., 2020), or between certain EV and recipient cell combination (Gasser et al., 2003; Chivet et al., 2014). In most of these studies, EV tropism was detected at the level of uptake (Jurgielewicz et al., 2020), however, it became evident that assessing EV functionality serves as a more effective physiological endpoint for detecting and understanding EV communication. Notably, our findings highlight that the matching of EV type and recipient cell is crucial for EVs to exert their specific functionality.

The unique downregulation of ZNF-81 in receptive endometrial epithelial cells upon JAr EV stimulation could be due to the presence of specific molecular cargo as luminal content or on the surface of JAr EVs. In our previous study, we demonstrated that JAr EVs carry a unique RNA cargo compared with HEK EVs (Godakumara et al., 2021). Notably, we found that the influence of EV RNA transcripts on modulating the target cell transcriptome was limited, suggesting a significant contribution of other EV biomolecules, particularly proteins. Therefore, we focused on exploring the proteomic cargo of JAr EVs in comparison to HEK EVs, considering it as a potential mediator of the effects of JAr EVs on receptive endometrial cells. These proteins may encompass enzymes, surface receptors, signaling proteins, and other bioactive molecules that may play a pivotal role in mediating the observed effects (Chen et al., 2021; Kim et al., 2021; Hánělová et al., 2024). As expected, there were substantial protein cargo differences between JAr and HEK EVs, which could further explain the observed tropism of JAr EVs towards receptive EECs. Functional enrichment and pathway enrichment analysis of the proteins distinctively found in JAr EVs showed pathways such as “ECM receptor-interaction,” “extracellular matrix organization,” “focal adhesion,” “cell adhesion,” and “adherens junctions.” These pathways have particular significance in the process of embryo implantation as well (Singh and Aplin, 2009; Godakumara et al., 2021; Poh et al., 2021; Liu R. et al., 2022). Among the proteins highly enriched in trophoblast analogue JAr EVs, compared to HEK-293 EVs, we found proteins linked to embryo-maternal communication, implantation, and early embryo development. FBLN2, ITGB4, BCAM, ITGB5 are some of the proteins vital in cell-cell adhesion that mediate the interactions between embryonic and maternal tissues, facilitating attachment, invasion, and completion of successful implantation (Chang et al., 2017; Meng et al., 2020; Ma et al., 2021; Bhattacharyya et al., 2022). During the peri-implantation phase, embryos undergo differentiation that is crucial for successful embryo implantation and further development. CLDN6, ADGRG1, EPCAM, FOLR1, ERVW-1 and APOD are some of the proteins observed in JAr EVs that has vital roles in early embryo development and differentiation (Moriwaki et al., 2007; Nagao et al., 2009; Strandgaard et al., 2017; C.West et al., 2022). In our study, we observed that MMP2 and MMP14 are among the highly enriched proteins in JAr EVs. Metalloproteinases (MMPs) and other proteolytic enzymes assist endometrial extracellular matrix remodelling (Cabral-Pacheco et al., 2020). This remodelling is critical for embryo invasion and implantation, enabling trophoblast to penetrate the endometrial epithelium establishing contact with endometrium.

Emerging evidence suggests that the EV protein cargo may change based on the cell of origin and metabolic status (Uzbekova et al., 2020; Hánělová et al., 2024). However, the bioactivity of natural protein cargo require further investigation. Therefore, we investigated whether any protein molecules in JAr EVs were responsible for ZNF81 downregulation in endometrial cells. We found multiple transcription factors (CTNNB1, HDAC2, and NOTCH1) that were specially enriched in JAr EVs compared to HEK EVs, which may be linked to ZNF81 gene control in endometrial cells. CTNNB1 is a critical transcription factor involved in various cellular processes, particularly the Wnt signalling pathway (Liu J. et al., 2022). Interestingly, embryonic CTNNB1 is required for priming the endometrium for implantation as well (Takezawa et al., 2023). NOTCH1 is involved in Notch signalling pathway and activates genes responsible for cell proliferation, differentiation, and apoptosis. NOTCH1 signalling is critical for embryonic development and the maintenance of adult tissue. Timely expression of Notch1 is critical for embryo implantation competency (Chu et al., 2011). HDAC2 is a crucial member of the histone deacetylase family and is involved in the regulation of gene expression via epigenetic modifications. HDAC2 is also known to play a role in embryonic development and implantation (Dsilva et al., 2023). Therefore we postulate that these transcription factors that govern ZNF81 gene regulation may also play a role in the embryo implantation process. However, further experimental evidence is required to confirm the bioactivity of these transcription factors in the endometrium.

Several hypotheses can be explored for understanding the mechanism of action of EVs on their target cells. One hypothesis is that EV surface proteins bind with receptors on receptor cells, inducing specific signalling pathways (Hoshino et al., 2015; Sushrut et al., 2017). Additionally, EV uptake inhibitors can be employed to inhibit EV fusion or internalization, allowing for a better understanding of the exact EV uptake pathways associated with specific functionality. Recombinant proteins for transcription factors can also be utilized to track EV cargo delivery and function (Liu Q. et al., 2022).

## 5 Conclusion

In conclusion, our findings highlighted that the downregulation of ZNF81 is a distinct response observed in receptive endometrial epithelial cells when exposed to trophoblast derived EVs. Moreover, we established that specific functional changes induced by EVs are, in part, associated with both the origin of the EVs and their protein cargo. Hence, evidence collected from the current study strongly supports the idea that EV specificity exists and is determined by a combination of factors, including the distinctive properties of EVs and the recipient cell type. This unique model of embryo implantation could be used to further decipher the exact mechanism of specific EV functionality.

## Data Availability

The data presented in this study are available in the ProteomeXchange Consortium via the PRIDE with the dataset identifier PXD050024.
